# Extracellular Vesicles in Sickle Cell Disease: Plasma Concentration, Blood Cell Types Origin Distribution and Biological Properties

**DOI:** 10.3389/fmed.2021.728693

**Published:** 2021-08-20

**Authors:** Elie Nader, Yohann Garnier, Philippe Connes, Marc Romana

**Affiliations:** ^1^Laboratoire Inter-Universitaire de Biologie de la Motricité EA7424, Team “Vascular Biology and Red Blood Cell”, Université Claude Bernard Lyon 1, Université de Lyon, Lyon, France; ^2^Laboratoire d'Excellence du Globule Rouge, PRES Sorbonne, Paris, France; ^3^Université des Antilles, UMR_S1134, BIGR, Pointe-à-Pitre, France; ^4^Université de Paris, UMR_S1134, BIGR, INSERM, Paris, France

**Keywords:** extracellular vesicles, sickle cell disease, inflammation, coagulation, oxidative stress, endothelial dysfunction

## Abstract

Prototype of monogenic disorder, sickle cell disease (SCD) is caused by a unique single mutation in the β-globin gene, leading to the production of the abnormal hemoglobin S (HbS). HbS polymerization in deoxygenated condition induces the sickling of red blood cells (RBCs), which become less deformable and more fragile, and thus prone to lysis. In addition to anemia, SCD patients may exhibit a plethora of clinical manifestations ranging from acute complications such as the frequent and debilitating painful vaso-occlusive crisis to chronic end organ damages. Several interrelated pathophysiological processes have been described, including impaired blood rheology, increased blood cell adhesion, coagulation, inflammation and enhanced oxidative stress among others. During the last two decades, it has been shown that extracellular vesicles (EVs), defined as cell-derived anucleated particles delimited by a lipid bilayer, and comprising small EVs (sEVs) and medium/large EVs (m/lEVs); are not only biomarkers but also subcellular actors in SCD pathophysiology. Plasma concentration of m/lEVs, originated mainly from RBCs and platelets (PLTs) but also from the other blood cell types, is higher in SCD patients than in healthy controls. The concentration and the density of externalized phosphatidylserine of those released from RBCs may vary according to clinical status (crisis vs. steady state) and treatment (hydroxyurea). Besides their procoagulant properties initially described, RBC-m/lEVs may promote inflammation through their effects on monocytes/macrophages and endothelial cells. Although less intensely studied, sEVs plasma concentration is increased in SCD and these EVs may cause endothelial damages. In addition, sEVs released from activated PLTs trigger PLT-neutrophil aggregation involved in lung vaso-occlusion in sickle mice. Altogether, these data clearly indicate that EVs are both biomarkers and bio-effectors in SCD, which deserve further studies.

## Introduction

Sickle cell disease (SCD) is one of the most frequent autosomal recessive genetic disorder that affects about 3.2 million people worldwide (1, 2). SCD is an umbrella term encompassing several sickle cell syndromes having in common the production of an abnormal hemoglobin, named hemoglobin S (HbS) instead of the normal hemoglobin A. HbS is produced as a result of a single base mutation (rs334) in exon 1 of the β-globin gene, leading to the replacement of a hydrophobic glutamic acid residue by a hydrophilic valine residue at the sixth position of the mature β-globin chain (3). Sickle cell anemia (SCA) results from the homozygous inheritance of the β^S^ mutation, whereas co-inheritance of β^S^ with other mutations such as β^C^, β^DPunjab^, β^OArab^ or β-thalassemia alleles lead to the other most frequently encountered sickle cell syndromes, namely HbSC, HbSDPunjab, HbSOrab and HbS-β-thalassemia, respectively, the latter one being subdivided in HbSβ^0^-thal and HbSβ^+^-thal (4).

Chronic anemia is a common clinical feature associated with the disease, as well as the occurrence of frequent and recurrent vaso-occlusive crises. In addition, SCD patients may exhibit various acute and chronic complications affecting a large number of organs such as the lungs, heart, kidneys, brain, skin and bones (5). Among the four drugs approved for prophylaxis and treatment of complications related to SCD, namely, hydroxyurea (HU), L-glutamine, voxelotor, and crizanlizumab; HU is the most commonly prescribed treatment (6). It is worthwhile to notice that SCD is characterized by a huge inter-individual variability in its clinical presentation, including for patients sharing the same sickle cell syndromes (7). This clinical variability could be related to the complex pathophysiology of this hemoglobinopathy for which new features and/or actors have recently been identified.

After a presentation of the main interrelated pathophysiological processes of SCD, we will present, in this review, compelling evidence showing that extracellular vesicles (EVs) are not only biomarkers of cellular activation and/or alterations occurring in SCD, but also bio-effectors able to modulate the different pathophysiological mechanisms.

## SCD Pathophysiology: A Complex Schema and Interrelated Pathways

HbS polymerization is the primary molecular event of SCD pathophysiology. In deoxygenated conditions, HbS proteins aggregate, form fibrous precipitates, and ultimately lead to red blood cell (RBC) sickling. These sickled RBCs are more rigid, fragile and therefore prone to disruption. HbS polymerization induces oxidative damage of the cytoplasmic membrane responsible for the stiffness of these cells and their shortened half-life (8–10). Increased RBC fragility and decreased deformability have been associated to chronic anemia and recurrent painful vaso-occlusive event, respectively (11). However, it has been recognized more than four decades ago that the transit time of RBCs in deoxygenated vascular areas, the territories affected by vaso-occlusive processes, would theoretically be too short to allow the sickling of RBCs (12). Activation and increased adhesiveness of various blood cell types such as neutrophils, monocytes and platelets to the endothelium (13–16), may trigger vaso-occlusion by decreasing blood flow and thereby increasing the RBC transit time in vascular bed with low oxygen content, leading to the sickling of RBCs before they can escape from the microcirculation (16). Sickled RBCs and stress reticulocytes, detected at abnormal level in the blood of SCD patients in response to anemia, also interact with endothelial cells (16). Aggregates of activated platelets and RBCs, monocytes or neutrophils, observed at abnormal levels in SCD patients (17–19), may also contribute to decreasing blood flow. The percentage of aggregates has been correlated with disease severity (20, 21). Finally, patients with the highest blood viscosity would also be prone to frequent vaso-occlusive crises because of the rise in vascular resistance and the slowing of blood flow (22–24).

### Pro-inflammatory State and Oxidative Stress

SCD has long been recognized as a chronic inflammatory disease associated with enhanced oxidative stress. A key role of intravascular hemolysis in these two conditions has been identified as shown and summarized in Table 1. In SCD patients and more particularly in those with SCA or Sβ^0^-thalassemia, hemolysis exceeds the capacity of plasma heme-binding proteins such as haptoglobin and hemopexin, leading to their depletion and thus the cell-free circulation of two toxic and oxidative molecules: hemoglobin and heme (29–31). Enhanced auto-oxidation of HbS induces the production of reactive oxygen species (ROS) such as superoxide anion, hydrogen peroxide and hydroxyl radical as well as the release of heme from sickle RBCs (25, 26, 42). Another significant source of ROS is related to the repeated episodes of ischemia-reperfusion occurring during repeated vaso-occlusive events and inducing high plasma levels of xanthine oxidase and NADPH oxidase (27, 28). Although conflicting results on antioxidant levels in SCD patients have been reported (43–45), the antioxidant capacity is insufficient to neutralize the excess of ROS, resulting in chronic oxidative stress (32). Enhanced oxidative stress may lead to endothelial damages through peroxidation of the lipid membrane and/or DNA fragmentation and ultimately cellular apoptosis (33) and has been linked to vascular alterations in SCD patients (34). In addition to these deleterious effects, ROS may promote vascular inflammation and NF-κB endothelial activation through the activation of redox-sensitive transcription factors such as (35). More recently, it has been shown that free heme may activate monocytes/macrophages (36, 37), neutrophils (38), platelets (39) and endothelial cells (40) inducing the secretion of pro-inflammatory cytokines and the activation of cell adhesion pathways, key events in heterocellular interactions leading to vaso-occlusion. Several studies have demonstrated that these heme-dependent cellular activations involved the Toll like receptor 4 (TLR4) and the NLRP3 inflammasome signaling pathways in endothelial cells and monocytes/macrophages (40, 46, 47). In addition, activation of neutrophils, one of the blood cell type playing a key role in vaso-occlusive process (16), by heme also induces the formation of neutrophil extracellular traps (NETs) for which high plasma concentration has been detected in SCD patients at steady-state with a further rise during crisis (38). NETs could participate to the chain of deleterious events occurring in SCD by promoting VCAM-1 and ICAM-1 endothelial expression (41), two proteins involved in the abnormal interactions between RBCs and endothelial cells (16), and by providing a scaffold for platelets, RBCs and pro-coagulant molecules (48).

**Table 1:** Involvement of intra-vascular hemolysis in oxidative stress and chronic pro-inflammatory state in SCD.

**References**	**Main findings**
(23, 25, 26)	Enhanced auto-oxidation of HbS leading to the production of ROS and hemolysis
(27, 28)	Repeated episodes of ischemia-reperfusion inducing high plasma levels of xanthine—oxidase, NADPH and ROS
(29–32)	Exceeding antioxidant capacity of the patient, including low levels of plasma heme and hemoglobin binding proteins such as hemopexin and haptoglobin leading to enhanced cell-free circulation of heme and hemoglobin
(33–35)	Vascular inflammation and endothelial activation mediated by ROS through the NF-κB pathway
(36–40)	Activation of monocytes/macrophages, neutrophils, platelets and endothelial cells by cell-free heme
(38, 41)	Production of neutrophil extracellular trap by activated neutrophils leading to higher endothelial expression of VCAM-1 and ICAM-1

### Decreased Bioactivity/Bioavailability of Nitric Oxide

Another deleterious effect of intravascular hemolysis is its impact on the bioactivity/bioavailability of nitric oxide (NO). NO, produced by endothelial NO-synthase, play a key role in the vascular physiology. This free radical induces vasodilation by relaxing perivascular smooth muscles, down-regulates the expression of endothelial adhesion molecules such as ICAM-1, VCAM-1, E- and P selectins and inhibits platelets activation (49, 50). Cell-free hemoglobin inactivates NO in a dioxygenation reaction leading to the production of methemoglobin and the release of heme into the plasma (51). This inactivation of NO is very efficient and 1,000 times faster than the one mediated by hemoglobin encapsulated into RBCs (52). Another consequence of hemolysis is the release of arginase by RBCs into the plasma, an enzyme that consumes plasma L-arginine, the precursor of NO, producing ornithine and urea, and thereby exacerbating the decrease of NO bioavailability (53). The decrease of NO bioactivity/bioavailability and thus the resulting endothelial/vascular dysfunction has been linked to a greater risk of developing several SCD complications such as pulmonary hypertension (54), legs ulcers (55), priapism (56), stroke (57) and proteinuria (58).

### Pro-coagulation State

Chronic activation of coagulation is another feature of SCD pathophysiology (59, 60). High plasma levels of markers of thrombin production such as prothrombin fragment 1.2 (F1.2), thrombin—antithrombin (TAT) complexes, D-dimers and plasmin—antiplasmin (PAP) complexes have been constantly detected in the plasma of SCD patients (61). Additionally, SCD patients exhibit low levels of protein C and protein S, two endogenous anticoagulants, presumably because of their chronic consumption related to ongoing coagulation activation (61). Tissue factor (TF), the primary initiator of extrinsic coagulation pathway, is one of the identified triggers responsible for the coagulation activation. In SCD patients, increased TF expression in monocytes (62), neutrophils (63) and circulating endothelial cells (64) have been detected. In agreement with the reported association between hemolysis marker levels and those of the coagulation activation in SCD patients (62), it has been shown that heme is able to promote TF expression in endothelial cells and blood mononuclear cells (40, 65). Moreover, the low levels of contact system proteins also suggest a contribution of the intrinsic pathway to thrombin generation (66). Externalized RBC phosphatidylserine (PS), detected in SCD patients in high amounts, may provide a negative charge surface allowing the docking of tenase and prothrombinase complexes, which in turn may promote the activation of the intrinsic pathway. Significant correlations between PS-positive sickle RBCs and plasma F 1.2, D-dimer and PAP complexes have been reported (67, 68). Additionally, high levels of cell-free DNA and nucleosomes released from neutrophils, two other triggers of the contact system activation, have been reported in the plasma of SCD patients (69–71). However, no correlation studies have been performed to our knowledge. If SCD chronic hypercoagulable state has been associated with an increased risk of limited complications such as venous thrombosis (72, 73), pulmonary hypertension (74) and *in situ* thrombosis of small vessels, it is worthwhile to notice that increased thrombin generation may also contribute to vascular inflammation (75).

This brief overview of SCD pathophysiology illustrates the fact that numerous abnormal pathways have been identified so far with multiple inter-relationships between these pathways. During the last decades, the involvement of the so-called extracellular vesicles in this complex pathophysiology has been documented.

## Extracellular Vesicles in SCD

### Classification of Extracellular Vesicles

**Figure 1:**
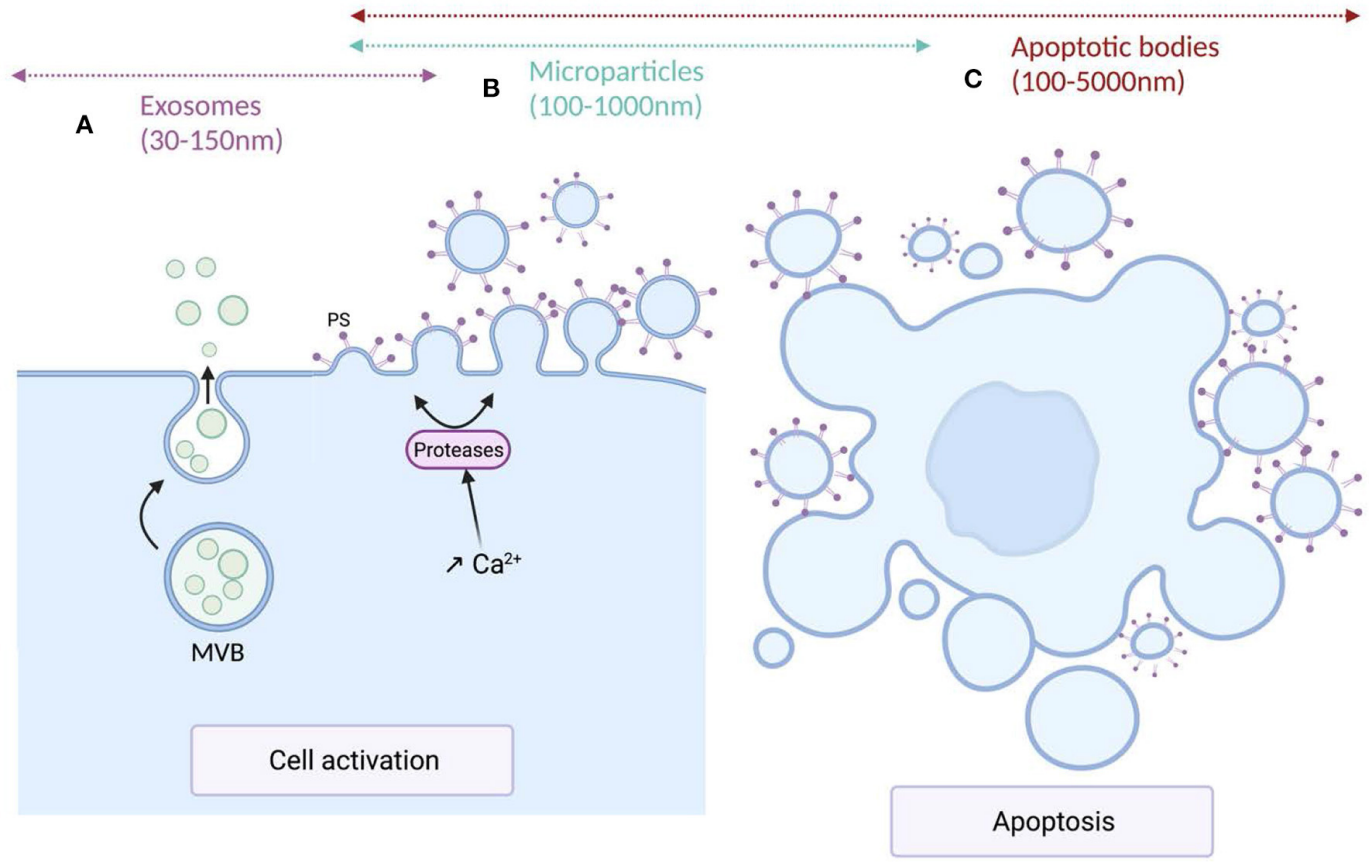
Mechanisms of production and size of the different extracellular vesicles types. **(A)** Exosomes are formed within multivesicular bodies (MVBs) and released upon fusion of MVBs with plasma membrane. Exosomes (sEVs) exhibit a narrow diameter ranging between 30 and 150 nm. **(B)** Microparticles (m/lEVs) diameter ranges from 100 to 1,000 nm. MPs derive from the cytoplasmic membrane of activated or apoptotic cells. Cell activation induces an increase of intracellular Ca^2+^ concentration leading to the translocation of phosphatidylserine (PS) to the outer leaflet of the cytoplasmic membrane and the activation of proteases that cleave the cytoskeleton, weaken its interaction with the cytoplasmic membrane, ultimately leading to the release of m/lEVs. **(C)** Apoptotic bodies are the largest EV subtypes exhibiting the wider size distribution (100–5,000 nm). They result from cell fragmentation and decomposition of the cell membrane of apoptotic cells.

Extracellular vesicles (EV) are a generic term for various particles delimited by a lipid bilayer, released from cells and detectable in numerous biological fluids (76). According to their genesis pathways, three main subtypes have been identified and named exosomes, microparticles (MPs) also called microvesicles, and apoptotic bodies. Exosomes, deriving from the endolysosomal pathways or from the outwards budding of the cytoplasmic membrane, are formed within the multivesicular bodies (MVBs) and released upon fusion of MVBs with plasma membrane (77). Compared to the other EV subtypes, exosomes exhibit a narrow size ranging from 30 to 150 nm in diameter. Microparticles, ranging from 100 to 1,000 nm in diameter, derive from the cytoplasmic membrane of activated, stressed or apoptotic cells. These conditions induce the increase of intracellular Ca^2+^ leading to the translocation of phosphatidylserine (PS) to the outer leaflet of the cytoplasmic membrane, a structural characteristic of these EV subtype, and to the activation of proteases that cleave cytoskeleton, weaken its interaction with the cytoplasmic membrane and ultimately allowing the release of MPs (78). Apoptotic bodies, the larger EV subtypes exhibiting the wider size distribution (100–5,000 nm) result from cell fragmentation and decomposition of the cell membrane of apoptotic cells (79, 80). The size distribution and the biogenesis pathways of the different EV subtypes are illustrated in Figure 1.

Over time, several techniques have been implemented for quantitative and/or qualitative analysis of EVs such as flow cytometry, dynamic light scattering, nanoparticles tracking analysis, scanning and transmission electron microscopy, cryo-electron microscopy and atomic force microscopy (81). Up to now, flow cytometry is clearly the most commonly used technique for EV analysis. Using fluorescent probes such as labeled Annexin V, a protein with high affinity for PS, and labeled antibodies directed against membrane proteins specific of each blood cell types, plasma concentration and cellular origin of EVs could be theoretically established. However, flow cytometry encounters several shortcomings including limited sensibility and resolution, leaving uncharacterized a significant proportion of the smallest EVs even with the most sensitive flow cytometers (82). Besides, it has been shown that numerous parameters such as technical characteristics of flow cytometers, pre-analytical and analytical conditions among others, significantly impact on both quantitative and qualitative EVs analysis. Since these pitfalls and limits have been extensively reviewed (83–85), they will not be discussed in the present review. To overcome these limitations, specific recommendations and guidelines have been produced (86, 87). However, most of the studies that aimed to characterize EVs in SCD, have been performed previously to their publication or did not apply these recommendations. Conflicting results found in the literature and described later are undoubtedly related to non-standardized procedures.

Originally described as dust cells in the late sixties (88), it has been demonstrated since then that EVs can mediate intercellular communication in both physiological and pathophysiological conditions (89–91) through the transfer to the recipient cells of their biological content, i.e., proteins, lipids, mRNA and miRNA (90, 92). In addition, high plasma levels of EVs have been detected in various diseases such as cardiovascular diseases, artherosclerosis, cancer and diabetes (93). All these disorders share several key pathophysiological components with SCD such as, increased risk of thrombosis, endothelial dysfunction, enhanced oxidative stress and high level of inflammation which may lead to increased release of EVs. For example, pro-inflammatory state and ischemic-reperfusion induce cellular activation and/or apoptosis and thus the production of EVs from various blood cell types such as endothelial cells, leukocytes, platelets and red blood cells as observed in ischemic coronary disease (94). In diabetic patients, endothelial dysfunction, evaluated by endothelial-dependent flow-mediated dilation, has been positively correlated with EV concentration released mostly by apoptotic endothelial cells (95). Altogether, these data strongly suggest that pro-inflammatory-state, pro-thrombotic-state and endothelial dysfunction are among the pathophysiological pathways associated with increased release of EVs.

It is important to notice that most of the reported studies in the field of EVs in SCD, do not provide any information on the biogenesis of these vesicles and used either their size and/or their density to classify them. Therefore, in the present review, we will use the terms of small EVs (sEVs) and medium/large EVs (m/lEVs), for exosomes and microparticles/microvesicles, respectively, according to the classification proposed by the International Society of Extracellular Vesicles (96). Knowing that there are significant overlaps of both size and density parameters between each EV subtype, this classification could be partly artificial and some of the reported associations and/or biological functions could be related to a mixture of different EV subtypes instead of one specific subtype.

### Plasma Levels of Extracellular Vesicles in SCD

#### In Steady State Condition

As indicated in Table 2, several reports have documented higher levels of EVs in SCD patients at steady state, i.e., at distance of acute complication and blood transfusion, compared to healthy individuals (97–102, 104, 105). Since most of these studies were performed using flow cytometers unable to detect sEVs, the previous observations are undoubtedly related to m/lEVS. Increased plasma concentrations of m/lEVs were detected not only in SCA patients but also in SC patients, although the levels reached in the latter population were not as high as in the former one (103). So far, no study specifically dedicated to patients with Sβ-thalassemia has been conducted. These m/lEVs derived mainly from platelets and RBCs while those originated from the other blood cell types such as endothelial cells, monocytes or granulocytes, were usually either barely detectable or detectable at low levels. In contrast, rather limited number of studies have been conducted on sEVs in SCD patients. Two reports from the same group described higher plasma concentration of sEVs in children and young adults with SCD at steady state level compared to controls (106, 107). The cellular origins of sEVs detected in SCD patients show a wider distribution than those of m/lEVs, being originated from RBC precursors, endothelial cells, lymphocytes, monocytes and platelets. All these plasma EV subtypes exhibit high concentrations in SCD patients, compared to controls, except for those originated from platelets. To our best knowledge, no study dedicated to apoptotic bodies has been performed in SCD yet.

**Table 2:** Comparison of blood cell type-derived EVs determined by flow cytometry between SCD patients and healthy controls.

**References**	**Type of EVs**	**Patients included**	**EV cellular origin assessed^a^**	**Compared to healthy controls**
Feng et al. (95)	m/lEVs	27 SCD patients	– RBCs (CD235a), – Platelets (CD41a), – Monocyte (CD14), – Endothelial cells (CD114)	– 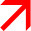 in SCD – 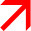 in SCD
Witwer et al. (96)	m/lEVs	50 SCD patients:	– RBCs (CD235a), – Platelets (CD41a),	– 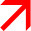 in SCD – 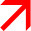 in SCD
Shet et al. (97)	m/lEVs	92 SCD patients:	– RBCs (CD235a), – Platelets (CD41a),	– 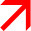 in SCD – 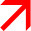 in SCD
Tantawy et al. (98)	m/lEVs	29 SCA patients	– RBCs (CD235a), – Platelets (CD41a),	– 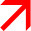 in SCA – 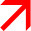 in SCA
Gerotziafas et al. (99)	m/lEVs	45 SCD patients	– RBCs (CD235a), – Platelets (CD61), – Monocyte (CD14), – Endothelial cells (CD106)	– 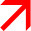 in SCD – 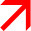 in SCD – 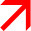 in SCD – 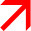 in SCD
Kasar et al. (100)	m/lEVs	138 SCD patients	– RBCs (CD235a)	– 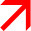 in SCD
van Tits et al. (101)	m/lEVs	232 SCA patients	– RBCs (CD235a), – Reticulocytes (CD71) – Platelets (CD61), – Leucocytes (CD45), – Endothelial cells (CD106)	– 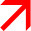 in SCA – 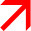 in SCA – 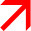 in SCA – 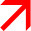 in SCA
Dembélé et al. (102)	sEVs	22 SCA patients	– RBC (CD235a), – Platelets (CD31/CD42b), – Monocytes (CD45/CD14), – Endothelial cells (CD309/CD133) – Progenitor cells (CD309/CD34)	– 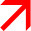 in SCA – 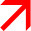 in SCA – 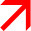 in SCA – 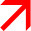 in SCA – 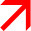 in SCA
Garnier et al. (103)	sEVs	33 SCD patients	– RBCs (CD235a), – Platelets (CD31/CD42b), – Monocytes (CD45/CD14), – Endothelial cells (CD309/CD133) – Lymphocytes (CD45) – Progenitor cells (CD309/CD34)	– 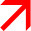 in SCA – 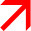 in SCA – 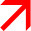 in SCA – 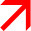 in SCA – 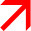 in SCA

a *In bracket is indicated the blood cell specific CD used to determine the cellular origin of EVs*.

The clinical severity of the disease has been linked to plasma EV levels measured at steady state. Severe vaso-occlusive phenotype (98, 104) and positive history of acute chest syndrome, pulmonary hypertension (98), osteonecrosis of the femoral head (108) and leg ulcers (102) have been associated with high concentration of m/lEVs released from various blood cell types. In contrast, lower concentration of reticulocyte-derived and RBC-derived m/lEVs were detected in SCA patients with a positive history of priapism and retinopathy, respectively, compared to SCA patients without these complications (102).

Few studies have specifically addressed the relationship between sEVs and the previous clinical course of SCD. Based on a cohort of 22 SCA children followed since birth and classified according to the painful vaso-occlusive rate, higher counts of sEVs originated from endothelial cells, progenitor cells, monocytes and lymphocytes were detected in the most severe patients compared to the milder ones (106). In addition, comparison of sEVs miRNA content between these two groups lead to the identification of miRNA expression patterns specific of the disease severity. A classification based on acute chest syndrome rate failed to detect difference in sEV levels except for those derived from monocytes (107).

Overall, if high levels of EVs have been repeatedly detected in SCD patients at steady-state, few of the associations with the severity of the clinical course of the disease have been reproduced and the usefulness of both EV subtypes as biomarkers of previous occurrence of specific SCD complication needs to be confirmed.

#### During Acute Complication

It has been shown that the clinical status of the SCD patients may impact m/lEVs concentration. Higher levels of m/lEVs have been reproducibly reported during vaso-occlusive crisis compared to steady state condition (97, 98, 100, 101, 109). However, the cellular origins of these vesicles, for which an increase had been detected, varied from one study to another. Several parameters may explain these discrepancies such as the study design, clinical definition of sickle cell crisis, the delay between blood sampling and hospital admission, and pre-analytical and analytical procedures used (110, 111). In the larger longitudinal survey published so far, in which 32 SCA patients were analyzed both at steady state and during painful vaso-occlusive crisis, a 2-fold increase in blood concentration of RBC-derived m/lEVs has been detected (112), in agreement with previous reports (98, 100, 109), as well as an increase of PS-externalization by these m/lEVs. Further studies are warranted to confirm and better describe these qualitative changes which impact on the biological properties of these vesicles, as described in subsequent paragraphs. To our knowledge, analysis of sEVs during the occurrence of SCD complications has not been performed yet.

#### In Patients Treated With Hydroxyurea

The impact of hydroxyurea (HU) treatment on the concentration of m/lEVs, the only EV subtype analyzed so far in relation to HU treatment, is still controversial. Indeed, decreased (98, 113, 114), increased (109, 115) and unchanged (96, 105) m/lEV concentrations have been reported in HU-treated SCA patients, compared to untreated patients. Knowing the wide distribution of m/lEV concentration in SCA patients (103, 112, 113), these contradictory results could be related to the cross-sectional design of these studies. In order to reduce the inter variability, we have implemented a longitudinal follow-up of SCA patients before and after 2 years of HU treatment (116). While no change in m/lEV concentration was detected, two qualitative parameters of m/lEVs originated from RBCs were modified during the course of HU treatment: EVs size was increased and their PS exposure was decreased. Such HU-related changes could affect their biological properties and need to be confirmed by further longitudinal studies.

### Triggering Pathways of EV Release in SCD

Several triggers and pathways of EV biogenesis in SCD pathophysiological context have been identified for two blood cell types: RBCs and platelets.

After the initial observation that repeated RBC sickling/unsickling induce the shedding of EVs (117), it has been shown that oxidative stress resulting from accelerated denaturation of HbS, leads to RBC membrane protein oxidation, weaker interactions between the membrane skeleton and lipid bilayer, destabilization of RBC cytoplasmic membrane and ultimately to EVs shedding (118–120). More recently, the involvement of hyperphosphorylation of Band 3, induced by the inhibition of SCD RBC tyrosine phosphatase (121, 122), in the release of EVs, has been documented. Indeed, direct relationship between tyrosine phosphorylation of Band 3 and the concentration of RBC-derived m/lEVs has been detected in SCD patients while *in vitro* inhibition of Syk kinases, the kinases responsible for Band 3 phosphorylation, was associated with lower shedding of these vesicles (123). Phosphorylation of Band 3 has also been linked to storage lesions of RBCs and RBC vesiculation (124). In addition, eryptosis, a condition associated with clustering of Band 3, induced by Band 3 phosphorylation, and characterized by increased RBC calcium level, PS externalization, RBC shrinkage, energy depletion and membrane blebbing, has been associated with the release of m/lEVs (125). Since Band 3, the most abundant protein of the RBC cytoplasmic membrane, plays a key role in membrane stability and deformability by linking the lipid bilayer to the cytoskeleton, it is not surprising that plasma level of m/lEVs produced by RBCs were reproducibly associated with both anemia level and hemolytic markers levels in SCD patients (103–105, 114, 126, 127).

Another identified biogenesis pathway of RBC-derived EVs relies on the dysregulation of the metabolism of sphingolipids. Sphingomyelinase, one of the key enzymes of this metabolic pathway, hydrolyzes sphingomyelin, a lipid representing 10% of the total lipid of the plasma membrane. It is activated by membrane curvature and increased mechanical bending stress in RBCs (128). The increased activity of sphingomyelinase in sickle RBCs from sickle mice compared to control mice, was linked to the generation of both sEVs and m/lEVs. Moreover, *in vivo* and *in vitro* pharmacologic inhibition of sphingomyelinase reduces their release from RBCs (129).

It has also been shown that infusion of thrombospondin-1, a protein stored in platelet α-granules and released upon activation, in mouse models, induces the shedding of RBC-m/lEVs through CD47 signaling pathway, a process exacerbated in sickle mice (130).

As previously described, several hemolysis end-products like cell-free hemoglobin and heme may activate platelets. The binding of HbS to GP1bα at the platelet membrane level induces a signaling process through Lyn, PI3K, Akt and ERK pathways and the shedding of m/lEVs (131). The binding of heme to TLR4, expressed not only by platelets but also by monocytes/macrophages, neutrophils and endothelial cells, activates these cells and presumably induces the release of EVs. Activation of these cells by inflammatory mediators such as cytokines, for which high levels are detected in SCD patients, may also lead to EVs shedding. In addition, *in vitro* experiments have shown that decreasing RBC oxidative stress, using the anti-oxidant N-acetylcysteine, was associated with lower shedding of m/lEVs by sickle RBCs (125). It is therefore tempting to hypothesize that ROS may also be involved in the release of EVs by these cells. However, no report has yet tested these hypotheses in SCD clinical context to our knowledge.

## Extracellular Vesicles as Bio-effectors of SCD

Since EVs exhibit biological properties, they could modulate, negatively or positively, pathophysiological SCD pathways. Some data suggest that vesiculation of cells could be a self-protective mechanism. Indeed, it has been shown that half of RBC-derived m/lEVs circulating in the plasma of healthy individuals were linked with natural antibodies directed against antigen-associated band 3 protein, a well-known senescence marker of RBCs, while a much lower fraction of RBCs was positive for this senescent marker (132). Based on these observations, it has been hypothesized that vesiculation may be a mechanism of removing damaged proteins from otherwise healthy cells and thereby increasing their lifespan. However, this phenomenon was described only for RBCs and may not be effective for the other blood cell types.

**Figure 2:**
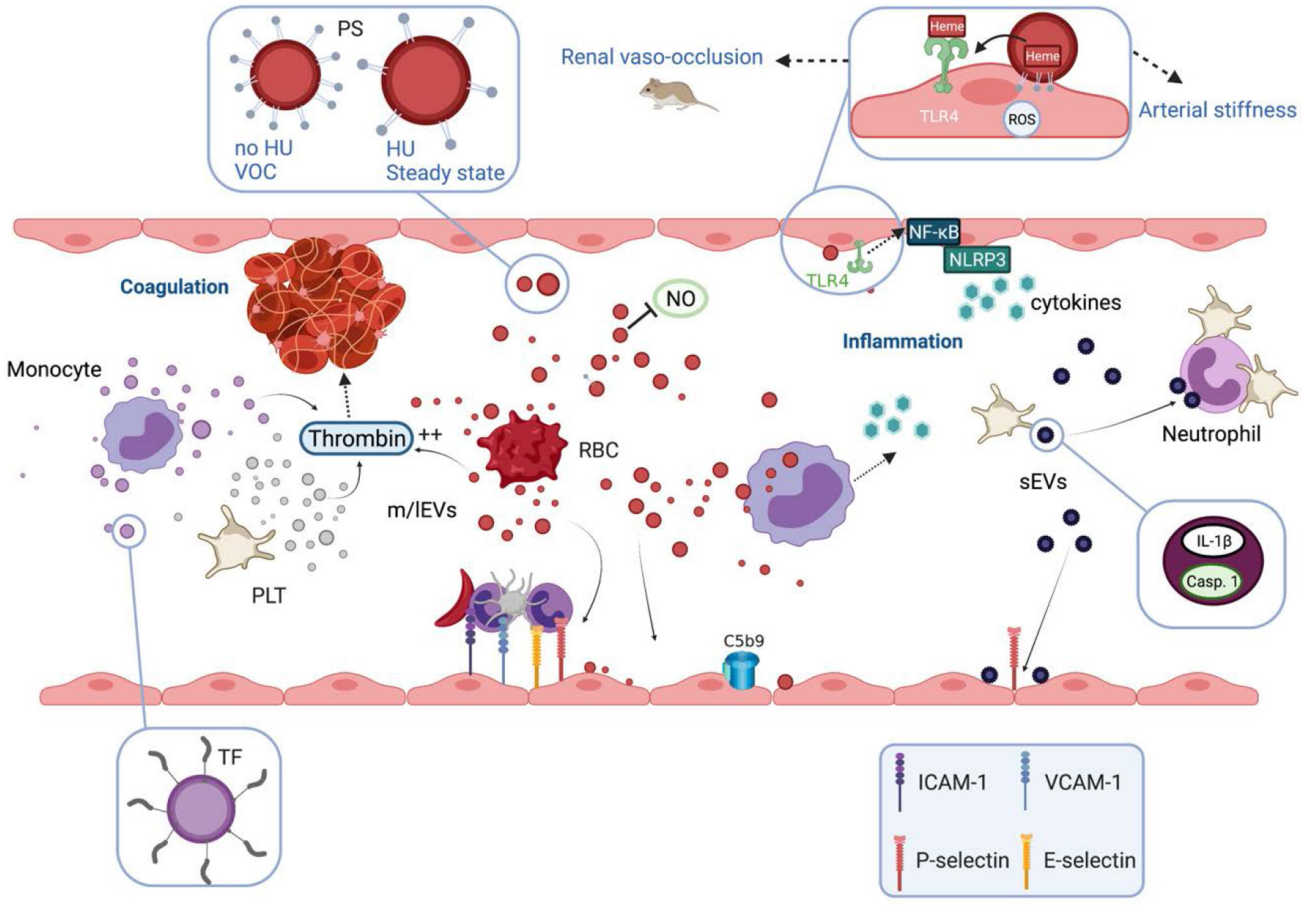
Biological properties and pathophysiological consequences of extracellular vesicles in sickle cell disease. Extracellular vesicles (EVs) partly cause the hypercoagulant and prothrombotic state known in sickle cell disease (SCD). m/lEVs generated *in vitro* from stimulated monocytes, RBCs or platelets are able to trigger thrombin generation through TF-dependent and TF-independent mechanisms. Intrinsic coagulation pathway activation by RBC- and platelet-derived m/lEVs relies on the exposure of phosphatidylserine at their outer membrane leaflet. EVs also contribute to the inflammatory state of SCD patients. m/lEVs produced *in vitro* from sickle RBCs can be internalized by monocytes leading to the secretion of several proinflammatory cytokines and can increase the adhesion of monocytes to the endothelium. m/lEVs generated *in vitro* by sickle RBCs have been shown to promote renal vaso-occlusion in sickle cell mice and to induce endothelial cell apoptosis and ROS production. The high level of PS exposed at the surface of these vesicles, as well as their content in heme, could play a role in their deleterious effects on the vascular function. RBC-m/lEVs directly isolated from SCD patients' blood samples, promote the expression of adhesion molecules (ICAM-1, E-Selectin) and the production of pro-inflammatory cytokines by cultured endothelial cells. The endothelial activation mediated by these EVs involves the TLR4 signaling pathway. These proinflammatory properties are considerably reduced for m/lEVs obtained from patients treated with HU, which exhibit low PS externalization. In contrast, m/lEVs collected from patients during vaso-occlusive crisis exhibit high PS exposure and have deleterious effects on endothelial cells. RBC-m/lEVs could decrease NO bioavailability through their scavenging effects. In addition, both externalized PS and heme exposed by RBC-derived m/lEVs obtained using a calcium ionophore, have been shown to activate complement system on endothelial cell membranes. In humanized SCD mice, the stimulation of platelets leads to the release of sEVs highly loaded with IL-1β and caspase-1, which bind to neutrophils and promote platelet-neutrophil aggregation. VOC, vaso-occlusive crisis; NO, nitric oxide; ROS, reactive oxygen species; HU, hydroxyurea; PS, phosphatidylserine; TF, tissue factor; TLR4, toll like receptor 4; Casp.1, caspase 1; PLT, platelets; RBC, red blood cells.

Besides this potential beneficial aspect of vesiculation, the involvement of EVs in several abnormal pathophysiological pathways of SCD has been documented, based on either association studies and/or direct testing of their biological properties in sickle mouse models or using *in vitro* experiments. These biological properties are summarized in Figure 2.

### Impact on Coagulation

EVs generated *in vitro* from stimulated monocytes, RBCs and platelets using calcium ionophore are able to trigger thrombin generation through TF-dependent or TF-independent mechanisms, when they are generated from the former and from the two latter cell types, respectively (133, 134). Accumulating evidence indicated that both activation pathways of coagulation are supported by EVs in SCD. The initial relationship detected by Setty et al. (68) in SCD patients between RBC-derived m/lEVs and plasma prothrombin fragment F1.2 has since then been confirmed and extended using other markers of coagulation activation (99, 126). In addition, RBC-m/lEVs of SCD patients were positively associated with acceleration in the propagation phase of thrombin generation while *in vitro* thrombin generation induced by these EVs was partly inhibited by anti-human factor XI (99). Activation of intrinsic coagulation pathway driven by RBC- and platelet-derived m/lEVs relies to the exposure of PS at their outer membrane leaflet which provides a suitable surface for the assembly of tenase and prothrombinase complexes (78). While platelet-derived m/lEVs are usually described as the most abundant m/lEVs detected in SCD patients, most of the studies detected association between RBC-EVs and coagulation activation. It is therefore temping to hypothesize that these unexpected results could be related to the higher density of externalized PS in RBC-m/lEVs than that of PLT-EVs (103) and therefore could activate coagulation more efficiently. In addition, it has been recently shown that m/lEVs released during red cell storage, can trigger coagulation activation not only through the canonical intrinsic pathway but also through the activation of a non-canonical pathway in which Kalikrein directly activates factor IX leading to thrombin generation (135). This observation also suggests that RBC-derived m/lEVs could be more efficient than platelet-derived m/lEVs in coagulation activation, but whether m/lEVs produced in the plasma of SCD patients exhibit similar biological properties remains unknown. In contrast to these studies, Shet et al. (97) reported positive relationship between TF-positive m/lEVS originated from monocytes and coagulation markers as well as the partial inhibition of the pro-coagulant activity of sickle m/lEVs by TF-neutralizing antibody in *in vitro* assays (97). Despite these discrepancies, these data clearly documented the involvement of m/lEVs in the hyper-coagulation and prothrombotic states, known to be significant contributors to vaso-occlusion in SCD (16).

### Impact on Pro-inflammatory Status

The capacity of EVs to interact with and to induce an inflammatory phenotype of several vascular cell types has been documented by several studies in the SCD context.

sEVs and m/lEVs produced *in vitro* from sickle RBCs, can be internalized by monocytes/macrophages leading to the secretion of several pro-inflammatory cytokines (129). In addition, peripheral blood mononuclear cells (PBMC) incubated with these EVs exhibited an increased adhesion to endothelial cells. Using m/lEVs produced *in vitro* by sickle RBCs, Camus et al. (130, 136) showed that their infusion in sickle mice promoted renal vaso-occlusion, reduced vasodilation of *ex vivo* isolated micro-vessels and induced endothelial cell apoptosis as well as ROS production (130, 136). The high level of PS externalized at the surface of these vesicles, as well as the fact that they contain a large amount of heme, would play a role in the impaired vascular function (130, 136). Both externalized PS and heme exposed by RBC-derived m/lEVs obtained using calcium ionophore, promoted alternative and terminal complement activation pathway in serum and on endothelial cell membrane (137).

Since content, structural characteristics and biological properties of EVs vary according to triggering factors (138, 139), we designed studies aiming at analyzing the biological properties of m/lEVs directly isolated from SCD patients in various clinical conditions. In these more pathophysiological relevant conditions, we have shown that m/lEVs isolated from patients at steady state induced ICAM-1 expression in cultured endothelial cells and thereby increased the adhesion of neutrophils (140). To decipher which blood cell type-derived m/lEVs are responsible for these biological effects, we used immuno-depletion to select vesicles according to their cellular origins and identified those released by RBCs as the main contributors (140). While pre-incubation of m/lEVs with annexin-V, a PS blocker, abolished the induced endothelial ICAM-1 overexpression, we have shown that the proinflammatory properties of m/lEVs collected during a vaso-occlusive crisis, a condition associated with high PS externalization of m/lEVs (114), were exacerbated. In contrast, these proinflammatory properties were considerably reduced for m/lEVs obtained from patients treated with HU, which exhibited low PS externalization (116). In addition, we presented evidence that the endothelial activation mediated by these EVs also involved TLR4 signaling pathway (125). In this study, we also detected a direct relationship between arterial stiffness in SCA patients and plasma concentration of RBC-m/lEVs. EVs could also disturb NO bioavailability through their NO scavenging effects (141). Altogether, these data strongly suggest that externalized PS, alone or associated with heme, or hemoglobin retained by these EVs, play a significant role in the induced endothelial cell dysfunction. However, the involved signaling pathways partly remain to be deciphered.

It has also been documented that sEVs, purified from SCD patients, could modify the phenotype of several vascular cell types. Indeed, Khalyfa et al. have shown that sEVs obtained from SCD patients exhibiting severe vaso-occlusive phenotype, decreased endothelial permeability, promoted P-selectin expression in endothelial cells, and induced a pro-adhesive phenotype of monocytes (106). However, the cellular origin of the vesicles responsible for these biological effects remains unknown. sEVs released from platelets may also play a significant role in the occurrence of acute chest injuries. In SCD humanized mice, a specific activation of platelets by LPS, an agonist of TLR4, leads to the activation of the NLRP3 inflammasome and to the release of sEVs highly loaded with IL-1β and caspase-1, which bind to neutrophils and promote platelet-neutrophil aggregation in lung arterioles (142). Such heterotypic aggregates may cause arteriolar microthrombi and mimic chest injuries observed in SCD patients.

## Future Directions

A recently described feature of EVs may be significantly relevant in SCD etiology and clearly deserves further studies. Fitzgerald et al. demonstrated that cytokines are not only released in soluble form, but also encapsulated in EVs in numerous biological fluids (143). They also showed that the relative fractions of free and EV-associated forms of cytokines are regulated and modulated upon cellular activation. EV-retained cytokines remained biologically active and could be released to their targeted cells by a yet uncharacterized mechanism. Since cytokines inside the vesicles are not detected by standard target cell-free assays and multiplexed immunoassays (143), the blood content of these inflammatory mediators has been underestimated in SCD patients so far, and the relationships between SCD clinical complications and cytokine levels are clearly issues which need to be reanalyzed. The quantifications of both free cytokines and those conveyed by EVs in SCD patients in various clinical conditions, could reduce this caveat and provide a better view of the inflammatory processes involved. Besides their cytokine contents, the other bio-active molecules contained in EVs, and specifically those produced *in vivo*, remain to be better characterized and/or to be identified. Indeed, very few studies have addressed this issue and the content of EVs reported so far has been mostly obtained from vesicles generated *in-vitro* using artificial experimental conditions. Future studies focusing on the description of *in-vivo* generated EVs characteristics are still pending. At last, the capacity of these EVs to modulate the phenotype of blood-cell types not yet investigated such as neutrophils, needs to be analyzed.

## Conclusion

In this review, we described the current knowledge regarding the quantitative and qualitative profiles of EVs in SCD patients, the clinical conditions modulating these plasma concentrations, the mechanisms involved in their genesis and their biological properties. While high plasma levels of EVs have been reproducibly described, uncertainties remain about their cellular origins. Nevertheless, these vesicles, both sEVs and m/lEVs, are able to modulate key processes of SCD pathophysiology and are therefore bio-effectors in SCD.

## Author Contributions

PC and MR conceived the concept and wrote the article. EN and YG edited and significantly improved the manuscript. EN drawn the figures. All authors contributed to the article and approved the submitted version.

## Conflict of Interest

The authors declare that the research was conducted in the absence of any commercial or financial relationships that could be construed as a potential conflict of interest.

## Publisher's Note

All claims expressed in this article are solely those of the authors and do not necessarily represent those of their affiliated organizations, or those of the publisher, the editors and the reviewers. Any product that may be evaluated in this article, or claim that may be made by its manufacturer, is not guaranteed or endorsed by the publisher.
